# Neurobrucellosis Presented by Cerebral Salt Wasting

**DOI:** 10.7759/cureus.8275

**Published:** 2020-05-25

**Authors:** Zeki Kemec, Cevat Tüzün, İdris Yıldırım

**Affiliations:** 1 Nephrology, Batman District State Hospital, Batman, TUR; 2 Radiology, Batman District State Hospital, Batman, TUR; 3 Internal Disease, Batman District State Hospital, Batman, TUR

**Keywords:** hyponatremia, brucella, cerebral salt wasting

## Abstract

This case reported here was a 27-year-old female patient, and she had no chronic disease other than hypothyroidism. She was brought to the ER with complaints of fever, chills, weakness, and hyponatremia. She has been diagnosed with brucellosis using a serum tube agglutination test (STAT). Cerebrospinal fluid (CSF) analysis and brain MRI findings supported the central nervous system (CNS) involvement of the brucella. Despite intense 3% NaCl hydration, her hyponatremia was persisting. Sodium stabilized on the 14th day when the antibrucellosis treatment effect was settled. Hyponatremia was attributed to cerebral salt wasting (CSW) due to neurobrucellosis.

## Introduction

Brucellosis, a zoonosis that arises from intracellular Gram-negative bacteria that belongs to the genus Brucella is endemic in Latin America, the Middle East, Iran and countries having the coast to the Mediterranean Sea and other countries [1-2]. Most of the infections are stemming directly or indirectly from being exposed to animals or ingestion of contaminated milk, cheese, and other animal products. Brucellosis is a multi-system infection that affects almost any organ and may present with a broad spectrum of clinical symptoms [3]. Central nervous system (CNS) involvement is the most severe and rare complication, and sometimes, it can be the only revelation of human brucellosis [4].

 Hyponatremia -- defined as a serum sodium concentration which contains less than 135 mmol/L -- is a common and important electrolyte imbalance that can be observed in isolation or, more often, as a complication of other medical illnesses (e.g., heart failure, liver failure, renal failure, pneumonia) [5]. Cerebral salt wasting (CSW) is seen in intracranial disorders, like subarachnoid hemorrhage, carcinomatous or infectious meningitis, and metastatic carcinoma, but in particular, after neurologic procedures [6]. The etiology of CSW is not fully understood. CSW is most generally observed after a CNS insult. 

 Our patient presented to the ED with complaints of dizziness, fever, and hyponatremia. CNS involvement due to neurobrucellosis was detected. Hyponatremia was linked to CSW. No other reason could be found in their resumes that might lead to this picture. Although we analyzed the literature extensively, we could not reach a similar case report. We present our unprecedented case. Thus, to our knowledge, this is the first case in the literature. In this case study, we present our unprecedented case.

## Case presentation

**Figure 1:**
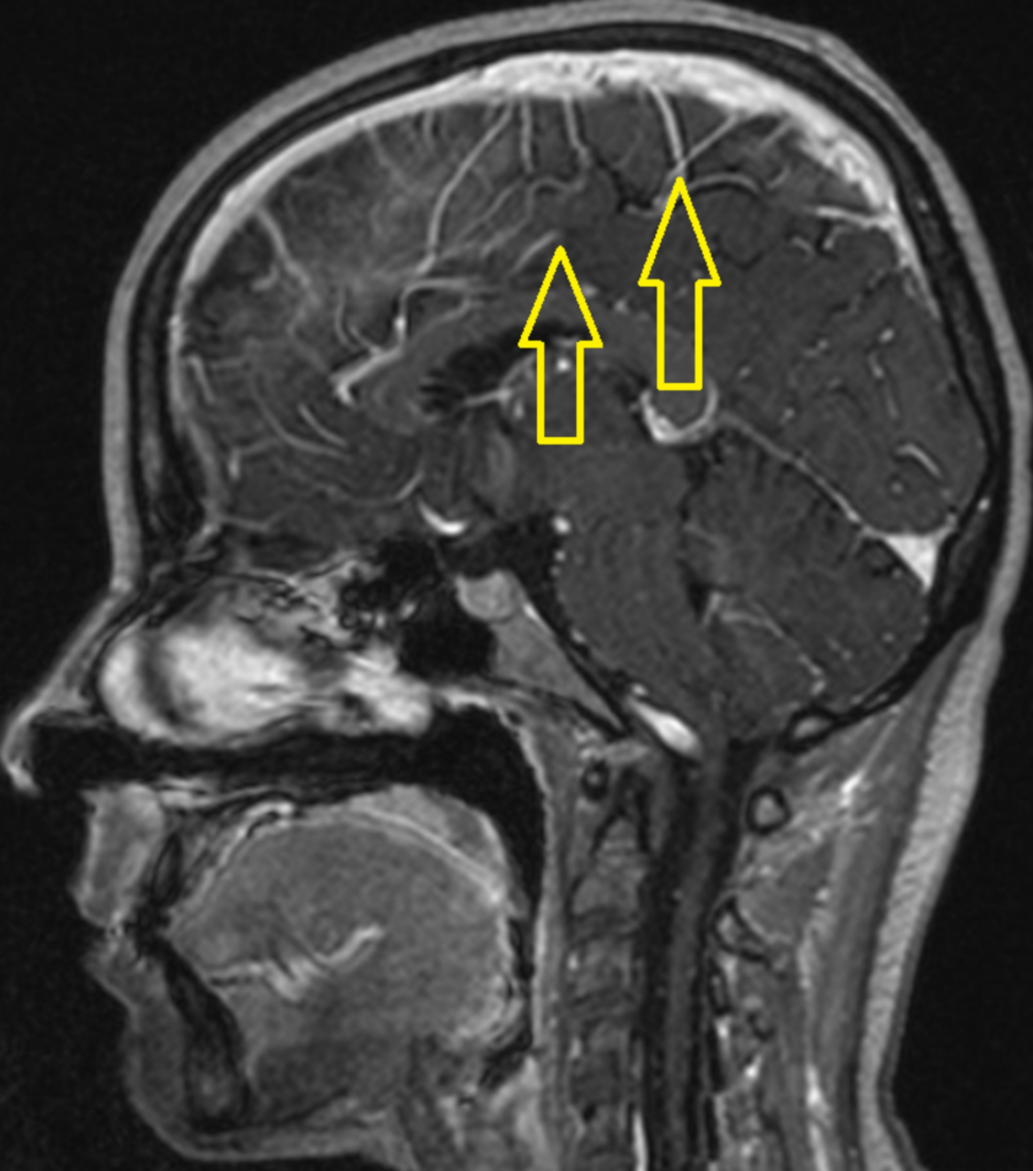
The sagittal post-contrast T1 weighted brain MRI image demonstrates leptomeningeal enhancement (yellow arrows).

**Figure 2:**
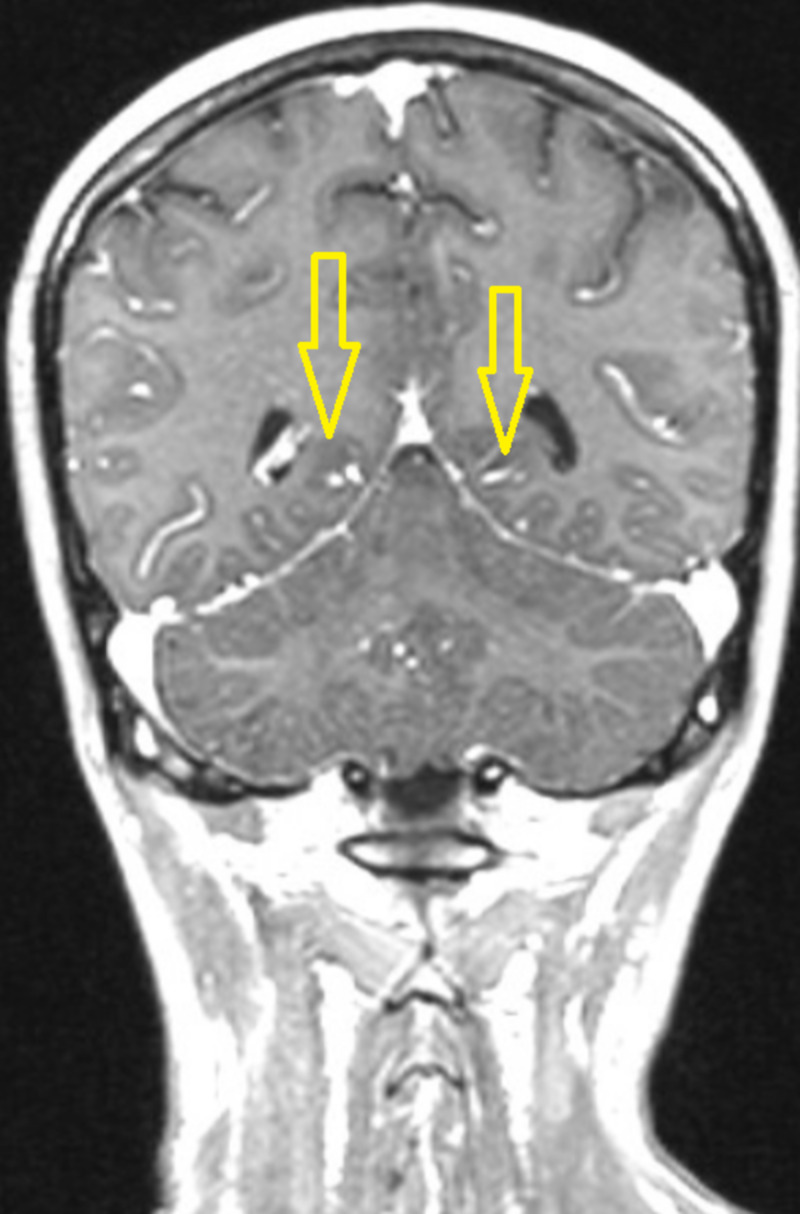
The coronal post-contrast T1 weighted brain MRI image demonstrates leptomeningeal enhancement (yellow arrows).

The patient is a 27-year-old woman, and the “informed consent” was obtained from herself. For five years, she has been suffering from hypothyroidism due to Hashimoto’s thyroiditis. The patient was prescribed a daily dose of 75 μg of levothyroxine (LVT) by an internal medicine specialist. Her checks were performed on a regular basis at the Internal Medicine Unit. During the follow-up, her thyroid hormones were monitored as normal. Two weeks ago, anorexia and muscle pain began. She ignored these complaints at first. She applied to our ED with complaints of fever, cold, chills, and dizziness that started three days ago. She was consuming fast foods. In the history of our case, conditions, such as kidney disease, chronic disease, and uncontrolled blood sugar, hypertension, infection, smoking, alcohol, medication (such as herbal medicine) and toxin use, were excluded. She was not complaining of diarrhea, dysuria, polyuria, and pollakiuria. She had no history of falling and head trauma. In physical examination, oral mucosa was dry, pulse rate 115/min (in sinus rhythm), blood pressure 90/60 mmHg, and fever was 38 degrees Centigrade. There was a state of vertigo and near syncope. Throat-urine-blood cultures were negative. Cardiac functions were normal on echocardiography. In her laboratory, hemoglobin (HGB): 9.5 g/dL, platelet (PLT): 61 × 109/L, sodium (Na): 117 mmol/L, creatinine (Cre): 0.44 mg/dL, aspartate aminotransferase (AST): 142 U/L, alanine aminotransferase (ALT): 56 U/L, direct bilirubin (DB): 0.33 mg/dL, indirect bilirubin (IB): 0.75 mg/dL, creatine phosphokinase (CK): 2122 U/L, chloride (CL): 84 mmol/L, calcium (Ca): 7.6 mg/dL, C-Reactive protein (CRP): 34.4 mg/L parameters were found (Table 1). Cardiac enzymes, HBV and HCV panel, coagulation parameters, procalcitonin, cortisol, and thyroid hormones were normal. For the diagnosis of brucellosis, serum agglutination tube test (STAT) with coombs antiserum was studied. Positive results were found at 1/640 titer. With the Elisa test, brucella IgM was positive and brucella IgG was negative. Anemia was detected in the blood count. Serum levels of parameters, such as vitamin B2, ferritin, folate, and transferrin saturation index, were normal. In peripheral blood smear, it was found normochromic normocyte erythrocytes, two-three thrombocytes in each area. Her leukocyte formula was normal. Anemia and thrombocytopenia were attributed to bone marrow depression of the brucella. Brucella treatment has been set. Ceftriaxone (4 g/day), rifampicin (600 mg/day), and doxycycline (200 mg/day) were started. A six-month treatment was planned. Antibiotics were administered in high doses due to neurobrucellosis. Previously used LVT treatment was continued at a dose of 75 μg. Profound hyponatremia (hypovolemic hypoosmolar-248.6 mosm/L) was not improved despite a 3% NaCl infusion. CNS involvement in brain MRI (Figures 1-2) was detected. In cerebrospinal fluid (CSF) examination after lumbar puncture, 179 leukocytes/mm3 were seen and 67% were detected as lymphocytes. CSF protein 111 mg/dL (15-45), CSF glucose 40 (45-80 mg/dL), simultaneous blood glucose 116, and CSF CL 113.6 mmol/L (122-131) were found. There were no oligoclonal bands; virus and tuberculosis polymerase chain reactions were negative. CSF culture was negative. Neurobrucellosis was considered. Urine sodium level was found as 210 mmol/L (normal urine sodium was <40 mmol/L). Urine osmolality is elevated above 100 mosmol/kg. Urine test and kidney ultrasonography were normal. She was diagnosed with CSW due to neurobrucellosis. On the 14th day of her follow-up, her sodium was corrected.

**Table 1:** Laboratory values of the patient. *The time period in which the patient is hospitalized. Cre: Creatinine, Alb: Albumin, Na: Sodium, K: Potassium, CL: Chloride, Ca: Calcium, AST: Aspartate aminotransferase, ALT: Alanine aminotransferase, TB: Total bilirubin, DB: Direct bilirubin, IB: Indirect bilirubin, LDH: Lactate dehydrogenase, GGT: Gamma-glutamyl transpeptidase, ALP: Alkaline phosphatase, CK: Creatine phosphokinase, P: Phosphorus, CRP: C-Reactive protein, WBC: White blood cell, HGB: Hemoglobin, RBC: Red blood cell, PLT: Platelet.

Parameters	Days of analysis				Reference values
	Day 1*	Day 7*	Day 8*	Day 14*	
Glucose (mg/dL)	116				70–110
Urea (mg/dL)	23				17–43
Cre (mg/dL)	0.44				0.7–1.3
Alb (g/dL)	31.8				35–52
Na (mmol/L)	117	125	133	137	136–146
K (mmol/L)	3.5				3.5–5.1
CL (mmol/L)	84				101–109
Ca (mg/dL)	7.6				8.8–10.6
AST (U/L)	142				0–50
ALT (U/L)	56				0–50
TB (mg/dL)	1.09				0.3–1.2
DB (mg/dL)	0.33				0–0.2
IB (mg/dL)	0.76				0–0.7
LDH (U/L)	908				0–248
GGT (U/L)	36				0–38
ALP (U/L)	48				30–120
CK (U/L)	2122				0–171
P (mg/dL)	1.2				2.5–4.5
CRP (mg/L)	34.8				0–5
WBC (×10^9^/L)	4.74				4.5–10.5
HGB (g/dL)	9.5				12–18
RBC (×10^12^/L)	4.29				4.2–6.1
PLT (×10^9^/L)	61				130–400

## Discussion

Neurologic symptoms of brucellosis reveal in 0%-25% of the patients and present along with chronic meningitis, meningoencephalitis, encephalitis, myelitis, myelopathy, stroke, paraplegia, radiculoneuritis, demyelination, intracranial hypertension, Guillain-Barre syndrome, subdural hemorrhage, abscess, cerebral venous thrombosis, cranial nerve involvement a merger of any of these symptoms and many levels of behavioral abnormalities [7]. CNS involvement in brucellosis is attributed to intracellular survival and the inflammatory response elicited by the organism gives rise to neuropathology [8]. Imaging abnormalities in neurobrucellosis are various and may imitate other infectious or inflammatory conditions that define inflammatory or demyelinating processes or a vascular insult and do not always correlate with the clinical situation [9]. Neurobrucellosis has neither a typical clinical situation nor specific CSF outcomes [7]. Examination of the CSF typically reveals increased protein absorption, a depressed glucose concentration, and a moderate leukocytosis composed mainly of lymphocytes [3]. Isolation of organisms from CSF is extremely difficult due to deficient organisms in the CSF. 1/640 titer found in STAT of our case with coombs antiserum STAT is easy to apply, has the advantage of low cost, and provides reproducible results and hence considered the gold standard serological method [10]. Brucella IgM was found positive. In CSF analysis, there was high protein and low glucose concentration. There was also leukocytosis, mostly composed of lymphocytes. There was CNS involvement in the brain MRI. When the patient's nutrition habit was questioned, it was learned that she was frequently fed with fast food (ice cream, ready-made milk, yogurt). The foods she eats are likely to be contaminated with Brucella. She was diagnosed with neurobrucellosis. Bone marrow depression, high level of transaminase, muscle enzyme, and CRP supported systemic brucellosis. High-dose antibiotherapy was started.

 Perturbation of sympathetic neural input into the kidney, which normally fosters salt and water reabsorption in the proximal nephron section through numerous indirect and direct mechanisms, might lead to renal salt wasting, resulting in decreased plasma volume. Plasma renin and aldosterone levels fail to rise suitably in patients with CSW despite a reduced plasma volume because of the disruption of the sympathetic nervous system. Furthermore, the release of one or more natriuretic factors could also take a role in the renal salt wasting observed in CSW. Volume exhaustion causes an elevation of plasma antidiuretic hormone (ADH) levels and impaired free water excretion [6]. Differentiating between CSW and syndrome of inappropriate ADH secretion (SIADH) can be difficult, as there is significant convergence in the clinical presentation. Vigorous salt replacement is required in patients diagnosed with CSW, while the fluid restriction is the treatment of choice in patients with SIADH. Infusion of isotonic saline to correct the volume depletion is generally efficient in conversing the hyponatremia in CSW as euvolemia will suppress the release of ADH. The disorder is, in general, transitory, with resolution arising within three to four weeks of disease start [11-12]. Since CSW was not well described and the diagnostic criteria are complex, the authors present simplified bedside criteria here [13] and the CSW was assessed in the existence of at least two out of four following features in a patient with hyponatremia: (1) clinical outcomes of hypovolemia, like hypotension, dry mucous membranes, tachycardia, or postural hypotension; (2) laboratory indication of dehydration, like elevated hematocrit, HGB, serum albumin or blood urea nitrogen; (3) negative fluid balance as determined by intake output chart and/or weight loss; (4) central venous pressure < 6 cm of water.

 Salt wasting nephropathy causing hypovolemic hyponatremia may rarely develop in a range of renal disorders (e.g., interstitial nephropathy, medullary cystic disease, polycystic kidney disease, partial urinary obstruction) with low salt intake [6]. The urinary sodium concentration of our patients was high. There was no cell and proteinuria in a urine test. Urinary ultrasound was natural. She had no kidney disease. The renal salt loss was not attributed to diseases of renal origin.

 Symptoms of hyponatremia range from nausea and malaise, with a mild reduction in the serum sodium, to headache, lethargy, decreased consciousness, and (if severe) seizures and coma. Plain neurologic symptoms most often are because of very low serum sodium levels (usually <115 mmol/L), resulting in intracerebral osmotic fluid changes and brain edema [6].

 The proposals for the treatment of hyponatremia depend on the existing understanding of the CNS integration to a change in serum osmolality. In the setting of an acute fall in the serum osmolality, neuronal cell swelling happens because of the water change from the extracellular space to the intracellular space (i.e., Starling forces). Therefore, the correction of hyponatremia should consider the limited capacity of this adaptation mechanism to answer to an acute change in the serum tonicity because the degree of brain edema and consequent neurologic symptoms depend as much on the rate and duration of hypotonicity as they do on its enormity [6]. The proposed treatment of acute hyponatremia differs by symptom gravity, as follows: mild to moderate symptoms, in patients at low risk for a herniation: 3% NaCl infused at 0.5-2 mL/kg/h [6]. Serum sodium concentration was monitored every 6 h of our case. Taking into consideration the neurological examination and its findings, 3% NaCl was administered in a controlled manner. The patient's hyponatremia was resistant to 3% NaCl (1.5 mL/kg/h) infusion. In the first three to five days, when the patient's 3% NaCl infusion was discontinued, the serum sodium concentration was decreasing to the range of 117-120 mmol/L. On the seventh day of hospitalization, when the high dose of the antibrusella treatment effect was settled, that is, when it clinically recovered (fever fell, joint pain and dizziness were stopped), serum sodium tends to increase (125 mmol/L). It was not like SIADH. The patient was hypovolemic and hyponatremic. Oral intakes were not good for three days. On physical examination, her body was dry. She had tachycardia and hypotension. She was having difficulty getting up. The patient's clinic, physical examination findings, STAT titer height, and brain imaging were supporting neurobrucellosis. The normalization of sodium when the antibacterial treatment effect was settled and the inefficiency of 3% NaCl alone were justifying neurobrucellosis-induced hyponatremia. Sodium deficiency has been attributed to CSW. In the literature, we could not reach the information that brucella could do CSW after CNS involvement. Other than that, there was no other reason that could cause hyponatremia. To our knowledge our case will be the first case in the literature in this respect.

## Conclusions

Cerebral salt wasting is a potential cause of hyponatremia in the setting of the disease of the CNS. Brucella is a systemic disease. It should be noted that there may also be CSW due to neurobrucellosis. The associated hyponatremia may be resistant to intense salt replacement. Physicians should be patient until the brucellosis treatment effect is settled.
